# Measuring Low Frequency Tilts

**DOI:** 10.6028/jres.098.014

**Published:** 1993

**Authors:** M. L. Kohl, J. Levine

**Affiliations:** Joint Institute for Laboratory Astrophysics, University of Colorado, Boulder, CO 80309-0440; National Institute of Standards and Technology, Boulder, CO 80303-3328

**Keywords:** borehole tiltmeter, pendulum, tilt tides

## Abstract

A borehole tiltmeter with a sensitivity of a few nanoradians is described. It is composed of two orthogonal horizontal pendulums with free periods of 1 s. The pendulums are insensitive to barometric pressure fluctuations, and the measured temperature coefficient is less than 30 nrad/°C. The range of the pendulums is about ±5 *μ* rad, and their response is linear within 1% and stable over several years. The performance of the tiltmeter in the field was evaluated using tidal data obtained from a closely spaced array of boreholes in Southern California. The long-term stability of the tiltmeter is generally better than 1 *μ* rad/yr. The data also indicate that instruments in boreholes at least 24 m deepare independent of surface effects. Several different capsules designed to couple the instrument to the surrounding material have been tested. In addition, an experimental method for estimating the magnitudes of local perturbation in the regional tilt field is described.

## 1. Introduction

Measurements of the deformation of the surface of the earth can be used to deduce information about the earth’s interior. One measure of the deformation is tilt which is defined as

$$\Omega_{D}=\hat{z}\times \left(s\times {\hat{d}}_{0}\right)/l,$$ (1)

where $\hat{z}$ is a reference direction and ***s*** is the relative displacement of a baseline of length *l* and direction ${\hat{d}}_{0}$ [1]. The reference direction, defined with respect to the earth’s surface, is usually taken as the local vertical. The displacement vector is a function of the strain tensor **E** the rotation vector ***r*** and the baseline measuring the deformation:

s=d⋅E+r×d. (2)

Vertical tilt is the deflection of an originally vertical baseline $\left({\hat{d}}_{0}=\hat{z}\right)$ while horizontal tilt is the vertical deflection of an originally horizontal baseline $\left({\hat{d}}_{0}\cdot \hat{z}=0\right)$. Near a horizontal free surface of an isotropic elastic body, normal stress vanishes and these two measurements are the same. There are two independent components of the tilt vector ***Ω***_D_, so measurements of the tilt along two orthogonal axes completely determine the tilt vector field.

The magnitude and frequency of tilt signals to be measured dictate the sensitivity and stability of instruments designed to measure them. Geophysical studies which use tilt data cover a broad frequency range. At one end of the frequency range are secular or long period tilts. This frequency range is of particular interest near fault zones where changes in the secular tilt rate could indicate changes in the status of the fault. Typical tectonic tilt rates for the western United States are on the order of 0.2 *μ*rad/yr [11]. An example of another frequency range which is investigated using tilt data is the tidal band around a few cycles per day. Baker[2] and Beaumont and Berger [3] predicted variations in earth tide amplitudes and phases due to changes in the elastic properties of surrounding material. Dilatancy near faults may lead to these changes. In order to measure the changes in tidal admittances and constrain models of the region, the tilt tides must be measured with a precision of a few percent. Typical amplitudes of the tidal components are on the order of 100 nrad. Therefore, the required sensitivity of a tidal tiltmeter is on the order of a few nanoradians.

## 2. The Instrument

The tiltmeter described here is a borehole instrument. It is composed of three parts—the sensor package, the electronics, and the frame. The sensor package contains the mechanical sensors which detect the tilt. The electronics quantify the movement of the sensor. The frame supports the sensor and the electronics so that the tiltmeter can be installed in a pressure-tight capsule which is lowered into a borehole.

### 2.1 Mechanical Parts

Since the tiltmeter was designed for borehole installations, the size of the sensor package is somewhat constrained, which in turn constrains the size of the pendulums. Because of this, the mechanical sensors used in the tiltmeter are horizontal pendulums. The advantages of using a horizontal pendulum are its compact size and mechanical amplification.

The amplification of a horizontal pendulum is calculated from its equation of motion. The equation of motion of an ideal horizontal pendulum with damping is

$$\ddot{\theta }+2\xi \omega_{0}\dot{\theta }+\omega_{0}^{2}\theta =\frac{g}{l}\Omega$$ (3)

where *θ* is the angular displacement of the pendulum, *ξ* is the damping coefficient, *ω*_0_ is the free period of oscillation of the pendulum, *l* is the length of the pendulum, and *Ω* is the tilt being measured (forced). (The expression changes slightly for a real pendulum because the radius of gyration is typically different than the distance from the pivot point to the center of the mass of the pendulum [1]). The amplification *A*(*ω*) of this pendulum is the ratio of the angular deflection of the pendulum to the applied tilt and is expressed as

$$A\left(\omega \right)=\frac{\theta }{\Omega }=\frac{g}{l\sqrt{{\left(\omega_{0}^{2}-\omega^{2}\right)}^{2}+{\left(2\xi \omega_{0}\omega \right)}^{2}}}.$$ (4)

For a horizontal pendulum, the free period of oscillation is related to the physical dimensions of the pendulum by $\omega_{0}^{2}=g$ sin *i/l*, where *i* is the inclination of the pendulum. Since the angular displacement of the pendulum is measured as a linear displacement of its free end, the mechanical sensitivity *G*(*ω*) of the pendulum is the ratio of the displacement of the pendulum to the applied tilt and depends on the length of the pendulum (*G*(*ω*)*=A*(*ω*)*l*, where *l* is the pendulum length).

The sensor package is composed of a mounting plate, two pendulums each with a pair of capacitor plates, and a cap which covers the mounting plate. It is nominally 7 cm in diameter and 6.5 cm in height. Boreholes with a 10 cm inner diameter are large enough to accommodate the package.

**Fig. 1:**
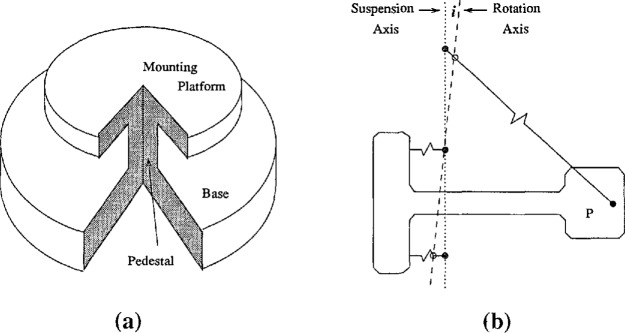
a) Schematic drawing of the mounting plate composed of the base and the mounting platform, and b) schematic of the horizontal pendulum, P. The effective axis of rotation of the pendulum (dashed) is inclined from vertical suspension axis (dotted) by the angle *i* = 2.3*°.* The pendulum is suspended on three springy wires (solid).

The plate on which the pendulums are mounted was designed to minimize the direct effects of temperature and barometric pressure (see Fig. 1a). It is machined from a single piece of stainless steel. The thick base of the plate provides good heat conduction. The mounting platform is machined above the base on a small pedestal. This configuration ensures that a pressure differential on the base results in an uplift of the mounting platform instead of a tilt. Two orthogonal pendulums (Fig. 1b) about 1 cm in length are each suspended between two capacitor plates attached to the mounting platform. The pendulums have a free period of 1 s and are approximately critically damped (*ξ* = 1) with air dampers. The plane of rotation of the pendulums is inclined from horizontal by about 2.3°. A cap covers the plate and the sensor package is hermetically sealed. The final package is effectively independent of barometric effects. The measured temperature coefficient of the sensor package is less than 30 nrad/°C. This is sufficient for borehole installations where the temperature stability at the bottom of a 30 m deep borehole is a few m°C/yr [1].

Tilt signals with frequencies lower than about 0.05 Hz (*ω*⪡ *ω*_0_) have an amplification independent of their frequencies. This is the low frequency amplification factor, which is equal to the cosecant of the angle of inclination. Secular and tidal tilt signals are in this low frequency range so the amplification factor for these signals is roughly 25. This corresponds to a mechanical sensitivity of 0.244 *μ*m/*μ*rad. The amplification of signals with frequencies between 0.05 and 10 Hz varies with frequency. In this regime, the form of the amplification transfer function is necessary to interpret data.

In order for the sensitivity to remain the same, the angle of inclination of the pendulum mustremain constant. The range of the pendulums is about ±5 *μ* rad which indicates that the angle of inclination can change by that amount. However, 5 *μ* rad is only a part in 10^4^ of the nominal inclination angle of 2.3°. Therefore, the sensitivity changes by less than 1% for the possible inclination angles of the pendulum.

**Fig. 2:**
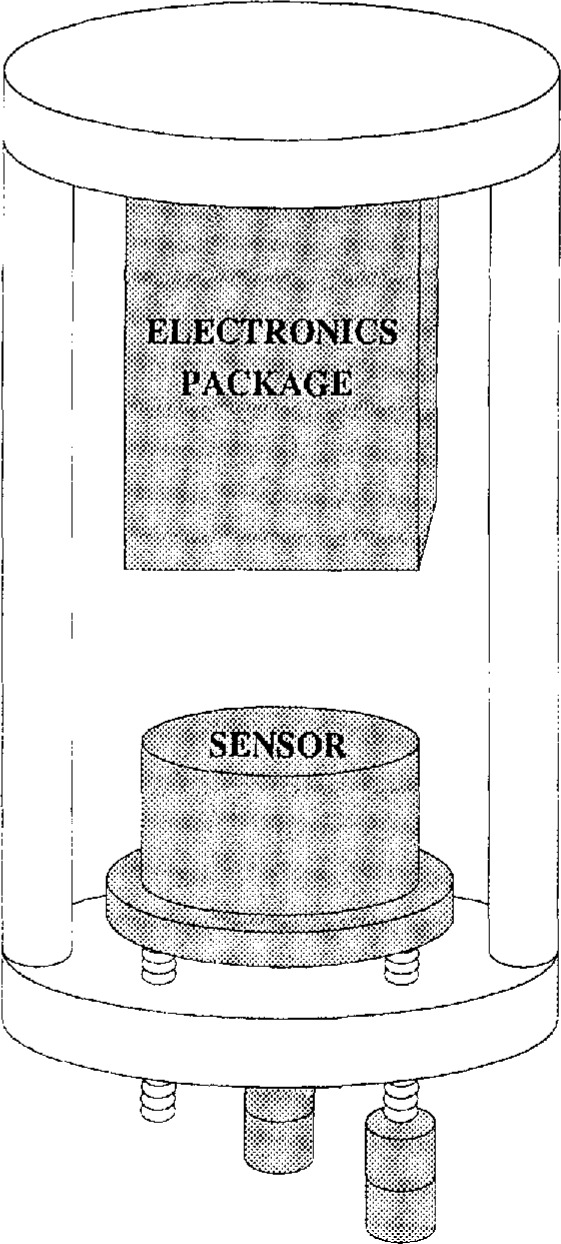
Drawing of the tiltmeter. The sensor is held on to three screws by a stiff spring. Two of the screws are motor driven and one is fixed.

The sensor package is held onto three screws in the tiltmeter frame by a stiff spring (see Fig. 2). Stainless steel ball bearings on the screws orient the package on a kinematic mount. Two of the screws are adjustable ultrafine leveling screws (3.15 threads/mm) and the third screw is fixed. Small dc motors with a 500:1 gearing ratio drive the movable screws so that the sensor package can be leveled after installation. The range of the leveling screws is about ±5°.

### 2.2 Electrical System

The displacements of the pendulum are determined using a capacitive transducer. Changes in capacitance are interpreted as displacements of the pendulum, so it is necessary to have any stray capacitances remain constant. Therefore, all of the electronics except the filter and data logger are mounted directly above the sensor package to minimize the length of the leads between the sensor and the electronics.

Each pendulum is suspended between two fixed capacitor plates so that the separation between the pendulum and the plates is about 1 mm. The plates have roughly 1 cm^2^ area, and the capacitance between the pendulum and either plate is about 2 pF. The displacement of the pendulum must be measured to about 0.5 nm to obtain the requiredsensitivity of a few nanoradians. This displacement will cause changes in capacitance of about five parts in 10^7^ which is equivalent to a 1 aF capacitance unbalance.

**Fig. 3:**
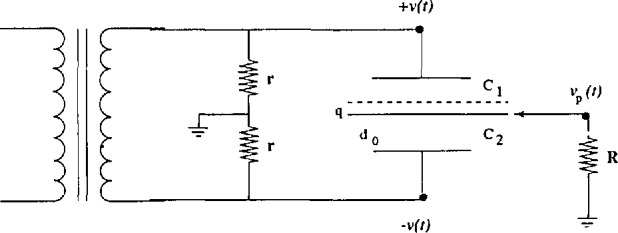
Schematic of the capacitive transducer. The separation between the pendulum and each capacitor plate is *d*_0_ ≃ l mm, and the displacement of the pendulum (center plate) from the center is *q*(*q*_max_*≃*10*^−^*^6^m). *R* is the input impedance of the detector.

We use an ac bridge circuit to measure the capacitance changes between the pendulum and its capacitor plates. A schematic of the bridge is shown in Fig. 3. The secondary of a transformer drives two matched resistors (r) to ground. This supplies the two outer plates with ac signals out of phase with each other. The pendulum and its capacitor plates form two arms of the bridge, and the other two arms are formed by the precision resistors.

The choice of drive frequency is controlled by two factors. In order to minimize the effect of 1/*f* noise, a high frequency is desirable. However, transformers provide the best frequency stability when operated below about 30 kHz. Also, higher frequencies limit the gain of the amplifier. We chose to use a frequency of 12.5 kHz.

The amplitude of the drive signal determines the sensitivity of the measurements. The larger the voltage between the plates, the smaller the minimum measurable displacement, since the rms signal-to-noise ratio of the system is increased. However, larger voltages increase the force of attraction between the pendulum and the plates. Therefore, the drive amplitude must be chosen so that the electrostatic force on the pendulum produces an acceptable nonlinearity in the displacements.

The force between the plates of a simple two-plate capacitor is expressed as

$$f=\frac{1}{2}\upsilon^{2}\left(t\right)\frac{\partial C}{\partial d_{0}},$$ (5)

where *υ*(*t*) *= V*_0_ sin *ωt* is the drive voltage on the plates, *C* is the capacitance between the two plates, and *d*_0_ is the separation of the plates. There are two components of *υ*^2^(*t*), one at twice the frequency of the drive voltage and the other a constant. The capacitor plates do not respond to the high frequencycomponent, so only the constant portion of the force is used to determine the displacing force on the plates. The electrostatic force between the plates of a simple capacitor becomes

$$f_{E}=\frac{1}{4}V_{0}^{2}\frac{\partial C}{\partial d_{0}},$$ (6)

where *V*_0_ is the amplitude of the drive voltage. For the three-plate arrangement of the tiltmeter, the capacitances between the pendulum and each capacitor plate are expressed as

$$C_{1}=C_{0}\frac{1}{1-\left(\frac{q}{d_{0}}\right)}$$ (7)

$$C_{2}=C_{0}\frac{1}{1+\left(\frac{q}{d_{0}}\right)},$$ (8)

where *C*_0_ is the capacitance between the pendulum and each plate when the system is symmetric, and *q* is the displacement of the pendulum from this position. The electrostatic force on the pendulum from the two fixed plates driven out of phase with each other is

$$f_{E}={\left(\frac{V_{0}}{d_{0}}\right)}^{2}C_{0}\frac{q}{{\left(1-{\left(\frac{q}{d_{0}}\right)}^{2}\right)}^{2}}.$$ (9)

To prevent this force from becoming very large, the pendulum must have stops so that *q*⪡*d*_0_. In this case, the displacement range of the pendulums is ±1.22 *μ*m corresponding to ±5 *μ*rad tilt.

The electrostatic force is balanced by the gravitational restoring force on the pendulum, so the energy balance equation for the system is

$$f_{E}\left(dx\right)=mgh,$$ (10)

where *dx* is the horizontal displacement of the pendulum, *m* is the mass of the pendulum, and *h* is the vertical displacement of the pendulum. The displacements of a horizontal pendulum of length *l* inclined by the angle *i* are

$$dx=l\cos\;i^{\prime }\sin\;\rho$$ (11)

$$h=l\;\sin i-l\;\sin i^{\prime },$$ (12)

where *i*′ is the angle that the displaced pendulum makes with the horizontal. When the pendulum is displaced by the angle *ρ*, *i*′ is related to *i* by

$$\sin\;i^{\prime }=\sin\;i\;\cos\;\rho .$$ (13)

The maximum capacitor plate drive voltage *V*_0_ is determined so that the additional displacement caused by the electrostatic force is less than one percent of the pendulum’s deflection [(*dx*)/*q* ⩽ 0.01)]. The value for *V*_0_ obtained by combining equations 9 and 10 is approximately

$$V_{0}≃\sqrt{\frac{0.01\;mgd_{0}^{2}\sin\;i}{2C_{0}l}}.$$ (14)

Since the mass of the pendulum is 1 g, the maximum capacitor plate drive voltage which will provide linearity within 1% is about 10 V. In practice, any nonlinearity in the system using a 10 V amplitude signal on the plates is not noticeable in either the calibration of the instrument or in the data. Typical drift rates of the instrument are about 1 *μ*rad/yr so most of the data are collected near the electrical center of the system where the effect ts of the nonlinearity are unmeasurable.

The output *υ_p_*(*t*)of the capacitive transducer is an ac signal whose amplitude is dependent on the magnitude of the displacement of the pendulum from the electrical center of the bridge and whose phase with respect to the driving signal indicates the direction of the displacement. It is calculated from Fig. 3 by noting that

$$\upsilon +\frac{i_{1}}{j\omega C_{1}}+\left(i_{1}-i_{2}\right)R=0$$ (15)

$$\upsilon -\left(i_{1}-i_{2}\right)R+\frac{i_{2}}{j\omega C_{2}}=0,$$ (16)

where *i*_1_ is the current flowing through *C*_1_and *i*_2_is the current flowing through *C*_2_. Using the definitions for the capacitances of the transducer with *q*⪡*d*_0_,the transducer output is

$$\upsilon_{p}\left(t\right)=\frac{V_{0}q}{d_{0}}\left(\frac{2R\omega C_{0}}{\sqrt{{\left(2R\omega C_{0}\right)}^{2}+1}}\right)\;\sin\left(\omega t+\phi \right),$$ (17)

where the phase angle *ϕ* is defined by

$$\tan\;\phi =-{\left(2R\omega C_{0}\right)}^{-1}.$$ (18)

For an ideal detector, the value of *R* is infinite, and the output amplitude would simply be *V*_0_*q*/*d*_0_. In our case, *R* is 10 M*Ω*, so the amplitude of the signal is about 94% of the ideal amplitude, with a phase angle of roughly −20°. The part of the signal which is in phase with the drive voltage to the outer plates has an amplitude of

$$V_{p}=\frac{V_{0}q}{d_{0}}\left(\frac{2R\omega C_{0}}{\sqrt{2{R\omega C_{0})}^{2}+1}}\right)\;\cos\phi .$$ (19)

When the outer plates are driven with an amplitude of 10 V, *V_p_* is about ± 10 mV for a capacitance unbalance of ± 1 fF(*q* = ±1 *μ*m).

A source follower is connected directly to the pendulum. It decreases the signal by about a factor of 2. The signal is then amplified in two stages with a total gain of about 1440 before entering the phase sensitive detector. The available output from the amplifier is about ±7.2 V corresponding to a capacitance unbalance of ± 1 fF, so that overloading of the amplifier is avoided.

The phase sensitive detector is composed of a JFET switch and an integrator. The switch is controlled by the same frequency that drives the outer plates of the transducer so that the integrator only works on the part of the amplified signal which is in phase with the drive frequency. The integrator has a dc gain of 10. The output of the phase sensitive detector is a dc voltage which is related to the transducer output by

$$V_{\text{out}}≃\frac{1}{2}V_{p}\times 14400÷2\pi ,$$ (20)

and is about ± 11.5 V for ± 1 fF capacitance unbalance.

The remaining electronics are located at the top of the borehole. The dc voltage tilt signals are sent through a single stage unity gain low-pass filter with about a 100 s time constant to average out microseismic background noise, whose spectrum peaks around a period of 6 s. The filtered signals are recorded on a datalogger at the site.

Each datalogger has eight channel capability, so tilt signals from up to four tiltmeters can be recorded by a single datalogger. The additional channels alternatively can be used to record other local data such as temperature, etc. The filtered signal is digitized with a 12 bit analog to digital converter for the voltage range ± 10 V, making the least count of the digitizer about 2.4 nrad for the nominal sensitivity of 2 V/*μ*rad. The digitized data are recorded every 6 min so that there are 10 data points per hour.

The datalogger is controlled with commands through the RS-232 interface which uses TTL protocol. This interface can be directly connected to a portable computer in the field, or to a modem for remote access. Data digitized and stored in RAM are retrieved each morning by an automatic dialing program on the computer in Boulder [6].

### 2.3 Calibration

The calibration of the tiltmeter is determined in the laboratory on a tilt table. It is conducted on a seismic pier in the basement of one of the buildings at the university. The pier is a large concrete block isolated from the building.

The calibration sweeps across the range of the tiltmeter sensor in approximately 1 *μ*rad steps at a rate of one step per minute. Because the table is driven by a screw, the steps are not instantaneous. Each step is completed within 20 s so that the pendulum has at least 40 s to stabilize before the corresponding voltage is determined. The overall sensitivity of the channels is set to be about 2 V/*μ*rad. Successive calibrations agree within 1% even when spaced several years apart [8]. The nominal calibration uncertainty is also about 1%.

Since changes in the amplifier circuits cause calibration changes, whenever any components fail in the field, the entire tiltmeter is replaced and the original tiltmeter is brought back to the lab for repair and calibration.

## 3. Installation and Alignment Methods

**Fig. 4:**
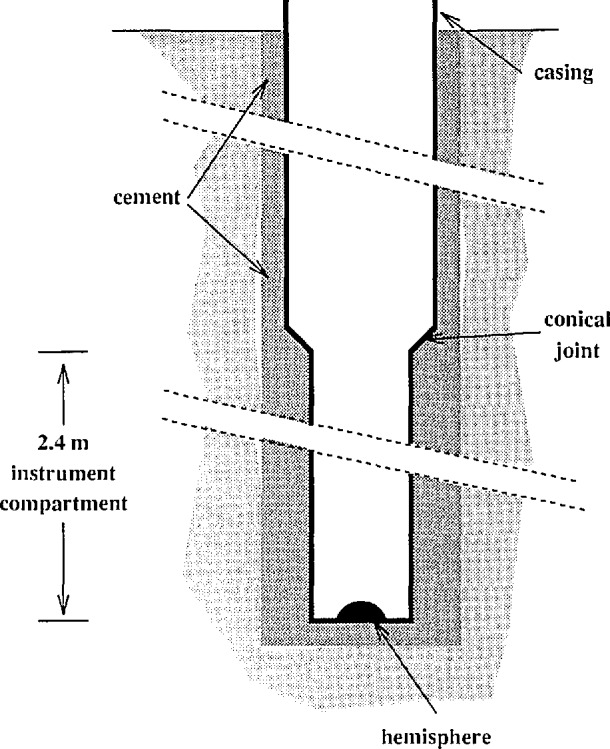
Schematic drawing of the boreholes (not to scale).

The boreholes are drilled by a conventional water well drill. They are lined with a steel casing as shown in Fig. 4. The holes are drilled slightly larger than the casing, since pressing the casing into the hole may lead to cracks in the casing. Because the tiltmeters have a leveling range of about ±5°, the lower section of the holes needs to be vertical within that range. When the drill is removed, concrete is poured down the hole so that the casing will be coupled to the surrounding material.

The lowest 2.4 m of the casing is stainless steel pipe with 10 cm inner diameter. A flat plate with a 1.3 cm diameter hemisphere in the center is welded to the bottom. The stainless steel instrument compartment is joined to 15 cm diameter carbon steel pipe. The joint is conical on the inside of the casing. As the casing is installed, additional lengths of 15 cm pipe are welded to form a continuous watertight pipe for the remaining depth of the borehole. Boreholes ranging from 15 to 120 m below the surface have been used.

Several different versions of the tiltmeter capsule have been designed. All versions are made from stainless steel pipe sections 2 m long. Their lids are pressure tight. The base of the tiltmeter frame is clamped in the capsule by three screws so that the sensor is located midway up the capsule. The differences between the versions is in the way that the capsule is located in the borehole.

**Fig. 5:**
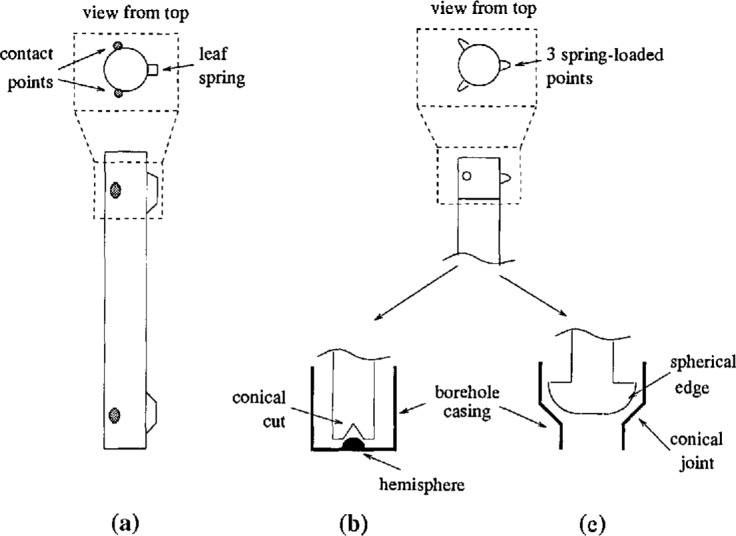
Three different borehole capsule designs. The original design (a) has a set of two contact points and a flat spring located at the top and at the bottom of the capsule. The other designs have three spring loaded pins located at the top of the capsule. The second design (b) centers the bottom of the capsule on the hemisphere in the casing while the third design (c) locates on the conical joing above the instrument compartment.

The original design [5] locates the capsule by referencing only the sides of the borehole (Fig. 5a). A flat plate is welded to the bottom of the capsule so that the weight of the capsule rests on the hemisphere in the bottom of the borehole. Near the top and the bottom of the capsule are two contact points and a flat spring 120° apart. The flat springs press the contact points against the inside of the borehole casing to couple the instrument to the surrounding material.

The second design uses a different technique for centering the capsule in the borehole. The hemisphere not only supports the weight of the capsule but also centers the bottom. A plate with a cone cut into it is welded to the bottom of the capsule. The cone centers the bottom of the capsule on the hemisphere at the bottom of the hole.,The top of the capsule is centered by three spring-loaded pins which are retracted by the weight of the capsule as it is being installed or removed. The capsule weighs approximately 133 N (13.5 kg mass), and each spring has about 26 N pressing against the casing when installed.

The third design is similar to the second, except that the bottom of the capsule is located on the conical joint above the instrument compartment. A plate with a spherical edge is welded to the bottom of the capsule. The top of the capsule is centered asbefore with three spring-loaded pins. Since the pins need a larger range of motion in order to press against the larger diameter casing, extra weight is added so that the pins retract completely during installation and removal. This capsule weighs about 267 N (27.2 kg mass), and the force on each spring is about 53 N.

The capsule is lowered into the borehole by a steel cable connected to the lid. Provided that the borehole is not too deep (less than 50 m), and the capsule is not too heavy, a capsule can be lowered by hand. For deeper holes and weighted capsules, a winch is used.

Once the capsule is lowered, it is important to know its azimuthal orientation. The drill bit wanders around the vertical direction when the boreholes are drilled, so direct observation is not possible except for very shallow holes (<10 m). Two different techniques were used to determine the orientation of the capsule depending on the depth of the borehole.

**Fig. 6:**
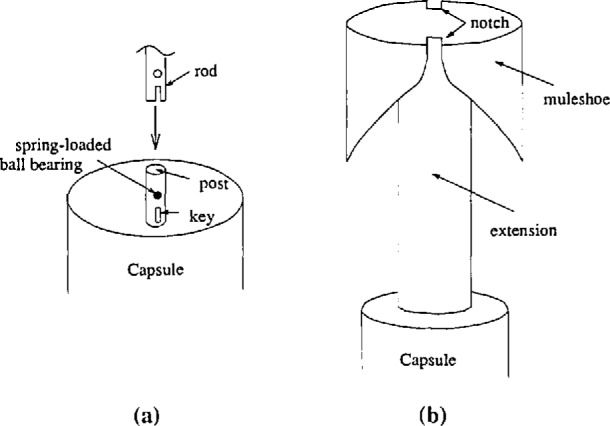
Methods for determining the orientation of the tiltmeters. Shallow holes use a series of rods (a) while deeper holes use a muleshoe attached to the top of the capsule (b).

Holes less than 50 m deep use a series of lightweight aluminum rods [5]. The lowest rod slides onto a post welded on to the lid of the capsule (Fig. 6a). It fits in only one direction and is held on by a spring-loaded ball bearing, so that the rods can be removed after the orientation of the capsule is determined. As the capsule is lowered, additional rods are added. Each rod is about 2.5 m long and will attach in only one direction to the rodbelow it. In this way, the orientation of the capsule is translated to the surface where the azimuth is determined using a compass.

The rods were originally calibrated outside of the borehole so that the accuracy of the measurement was a few degrees. Using alignment rods to determine the orientation of a capsule has several disadvantages. First, the alignment rods must be used every time that a tiltmeter is installed. Second, the rods, being made of aluminum to minimize their weight, are relatively easily bent. This may cause degrading of the azimuth measurement for each successive installation. Finally, the system is feasable only for shallow boreholes—the rod stiffness decreases with length, and the additional weight becomes appreciable.

For deeper holes a different technique was developed. The lid of the capsule was modified to include an extension with a muleshoe attached to the top (Fig. 6b). The muleshoe is composed of two shovel-like pieces pointing down the borehole. As the capsule is lowered, the shovels reach two keys welded to the inside of the casing. The shovels guide the capsule into the hole by referencing the keys. When the capsule is at the bottom of the borehole, the keys fit into two notches in the muleshoe. The orientation of the keys was determined by attaching a gyroscope to the muleshoe. The gyroscope orientation was measured several times with a repeatability of 2°. In general, oncethe orientation of the keys is known in a borehole, subsequent installations of that borehole need only note the orientation of the sensor with respect to the muleshoe. Since the muleshoe has two shovels, the amount of rotation of the capsule at the bottom of the borehole is reduced, however, there is a 180° ambiguity in the device. The ambiguity is easily resolved by looking at the tidal signals.

## 4. Performance in the Field

The tiltmeters have been installed in various locations during the past 12 years. The sites have included boreholes in Colorado [8], Wyoming [9], and California. All of the data presented here are from the California array, which consists of three closely spaced boreholes (within a radius of about 35 m) at different depths (24, 36 and 120 m).

**Fig. 7:**
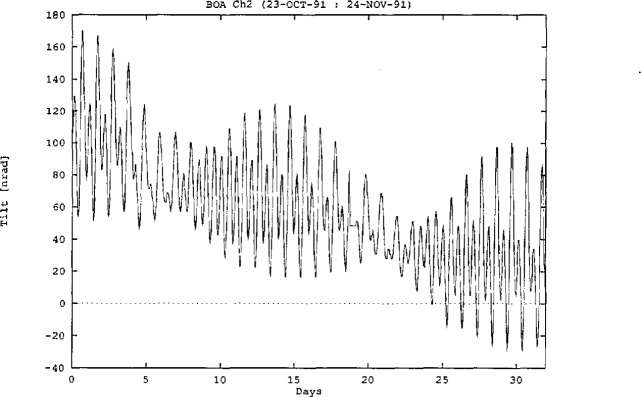
Typical time series from a 24 m borehole.

Figure 7 shows a typical tilt time series recorded from the 24 m borehole. The time series shows a general trend (about 1 *μ*rad/yr) as well as tidal components (amplitude about 10 nrad) with frequencies around one and two cycles per day. The trend in the data is a measure of the secular tilt present at the particular site combined with the instability of the instrument and the installation. For boreholes in Southern California, the expected secular tilt is less than 1 *μ*rad/yr, so the recorded trend in Fig. 7 is unlikely to be entirely attributable to secular tilt. Drift rates as low as 0.2 *μ*rad/yr have been observed.

**Fig. 8:**
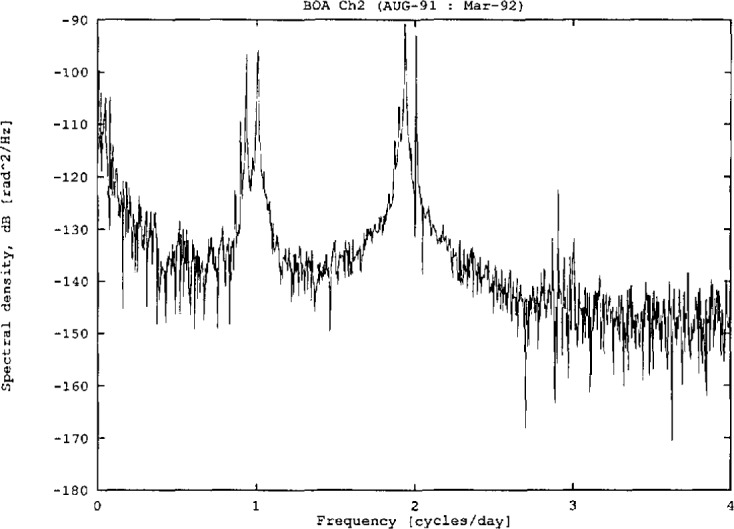
Power spectral density of data from the 24 m borehole.

Figure 8 is the power spectral density of a longer time series from the same borehole. The long period trend was removed from the time series before the spectrum was calculated. The signal to noise ratio at tidal frequencies is estimated by comparing the tidal amplitude to the inter-tidal noise amplitude. For these data, the signal-to-noise ratio for the larger components is 36–42 dB, which is typical of the data from California.

In order for geophysical interpretations of the tilt data to be accurate, it is important to understand how the measurements are affected by surface effects, cavity effects, and the existence of local inhomogeneities. Surface effects result from the measurement’s being made near the surface of the earth. Cavity effects arise from the presence of the instrument itself. Inhomogeneities near the instrument cause strain field distortions which can affect the local tilt field. The influence of each of these effects on our borehole tiltmeters has been investigated.

The proximity of the earth’s surface affects tilt measurements in several ways. Meertens and Wahr [10] showed that the local topography causes coupling of the strain field into the tilt field. The amount of coupling is dependent on the slope of the topography and can be calculated for any site. Temperature and barometric pressure may also affect the tidal tilt measurement due to thermal expansion and surface loading [7].

Data from the California array have been analyzed to estimate the size of each of these surface effects. The topography at the site is inclined by about 2.5° toward the southwest, so the topographic correction to the data is small and identical for each of the holes. The effect of the temperature and barometric pressure is estimated from the coherence spectra between the meteorological and tilt data. The data only show significant coherence amplitude for the barometric pressure and the tilt data from the 36 m deep hole. The coupling coefficient in this case is 7 × l0^−3^nrad/Pa at nontidal frequencies. Assuming that the coupling is independent of frequency, and since the measured barometric pressure tides have maximum amplitudes of around 60 Pa, the modifications of tidal admittances due to barometric coupling are less than 5%. Because the sensor package is independent of atmospheric pressure changes, the pressure induced tilts are likely to be caused by indirect effects. These effects are true tilts that arise from the spatial variation in the barometric pressure load.

The measured tilt data can also be affected by the presence of the instrument itself. The existence of a cavity causes strain field distortions which may produce anomalous tilting of instruments inside the cavity. This anomalous tilting results from either tilting of the cavity walls or differential motion between the tiltmeter capsule and the cavitywalls. Harrison [4] calculated the effect of distortions of the walls on tilt for several cavity shapes and found that a borehole tiltmeter will have no cavity effects when the sides of the borehole are referenced and only a small effect when the center of the bottom of the borehole is used as a reference. This result inspired the original capsule design which only references the sides of the borehole.

The size of the cavity effect arising from differential motion between the cavity and the capsule for the original capsule design is determined by comparing data obtained from multiple installations of a single borehole. Table 1 contains the amplitudes and phases along 0° and 90° azimuth ofone of the largest tidal components, M_2_(frequency ≃ 1.93 cycles/day), for different installations in a 24 m deep borehole. The phase is measured with respect to the local gravitational potential where a negative value indicates a phase lead. The values in parentheses are the uncertainties of the amplitudes and phases. Consecutive estimates of the tidal components during one installation are stable within 2%over several years. All of the data in this table were obtained with a single tiltmeter. The first two installations used the original capsule design installed along different azimuths. The first installation had the pendulums aligned with 15° and 105° azimuth, while the second installation was rotated to 314° and 440° azimuth. The differences betweenthe two installations is somewhat larger than expected from the performance of the tiltmeter during a single installation. Since measuring the tilt along any two orthogonal azimuths is sufficient to completely determine the local tilt field, these discrepancies indicate differential motion between the borehole and the capsule.

**Fig. 9:**
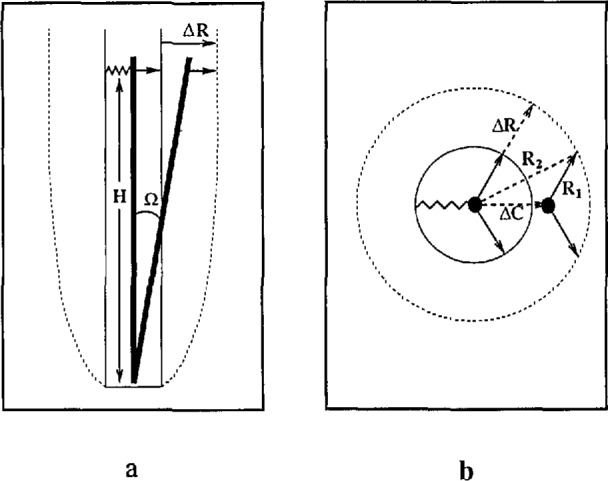
Tilt caused by uniform expansion ∆*R* of a borehole for a (a) two dimensional and (b) three dimensional hole. The capsule references the sides of the hole by a spring and contact points (arrows). The contact points remain a fixed distance from the sides of the hole, while the spring expands.

Subsequent study of the original capsule design revealed the source of this motion. Although the capsule references only the sides of the borehole, the reference points are asymmetric (two rigid contact points and one spring). This results in a cavity effect as shown in Fig. 9 because the lower set of reference points is located close the the bottom of the borehole. An applied strain field produces a tilt of the capsule, so the strain field is coupled into the tilt field along the spring azimuth. The magnitude of the coupling approaches 10% of the strain which is sufficient to account for the differences seen in the data. The second design (which eliminates the asymmetry of the reference points) was installed in the borehole in March 1992. At this point, there has only been one installation of the new capsule, so it is unclear whether the repeatability between installations has been improved.

The presence of local inhomogeneities near the tiltmeter can also influence the tilt data. This arises from the fact that the tiltmeters measure the tilt field using a relatively short baseline (2 m). Local disturbancesinthetiltfieldwithwavelengthsshorter than 2 *m* will be averaged out while longer wavelength phenomenon will be recorded. The magnitude of this effect is estimated by comparing data from the different boreholes.

Table 2 contains the M_2_ amplitudes and phases along 0° and 90° azimuth for each of the three boreholes. The data show marked differences between the boreholes which exceed calibrationuncertainties (1–2%), the long-term stability (2%) and the expected differences from measuring at different depths (<1%). The material properties at the site change between a depth of 15 and 35 m due to the presence of a weathering layer associated with the water table [12]. Investigation of the effect of the weathering layer on tilt data shows that the regional strain field can become coupled into the tilt field. The magnitude of the tilt-strain coupling depends on several factors including the distance between the layer and the tiltmeter.

The third capsule design was installed in the 36 m borehole to experimentally determine if there is a variation of material properties near the bottom of the hole. Table 3 lists the measured M_2_ admittances using the original capsule design and the third capsule design. The phase difference along 90° azimuth between the first two installations results from the asymmetry of the reference points as described earlier. Data from the third installation show a phase shift of about 26° along 0° azimuth from using symmetric reference points and changing the depth of the tiltmeter by only 2.4 m. This phase difference exceeds the maximum 10% strain coupling which could result from changing the reference points. Using reasonable estimates of the change in material properties, these measurements constrain the location and size of the weathering layer. The parameters obtained in this way are consistent with direct measurements using well logs and other data.

## 5. Conclusion

A geophysical borehole tiltmeter which has a sensitivity of a few nanoradians has been described. The stability of the calibration is less than 1% over several years. Consecutive estimates of the tidaladmittances are stable to 2% for any installation of the tiltmeter. The long term drift of the tiltmeter in the field varies between 0.2 *µ*rad/yr to more than 1 *µ*rad/yr.

Several items can be noted from the analysis of the California data which should be considered carefully when planning future borehole tiltmeter installations. First, knowledge of the orientation of the tiltmeter is useful for comparisons between different boreholes. Of the two methods described here, determining the orientation using a gyroscope is more desirable, since the uncertainty in the measurement is about ±1° and does not degrade with time. Second, tiltmeters installed at depths of at least 24 m show no evidence of surface temperature effects. This is also true for barometric pressure loading, provided that the sensor is installed far from any boundary layers such as a water table. A drilling log can provide useful information for determining the optimal depth of installation of a tiltmeter in the borehole as well as for interpreting the tilt data. Once a borehole is drilled, changing the location of the tiltmeter in the borehole can be a very sensitive technique for determining the existence of a local perturbation in the tilt field. Finally, the design of the tiltmeter capsule determines the amount of tilt-strain coupling in the instrument itself. We have found that asymmetrically referencing the sides of the borehole near the bottom can produce almost 10% tilt-strain coupling.

## Figures and Tables

**Table 1:** M_2_ data from multiple installations in a 24 m borehole. The phases are measured with respect to the local tidal potential (negative values are phase leads)

		0° azimuth		90° azimuth	
Data interval	Capsule type	Amplitude (nrad)	Phase (deg)	Amplitude (nrad)	Phase (deg)
Jun. 88–Aug. 91	original	18.44 (0.34)	124.5 (0.6)	18.48 (0.23)	−82.8 (0.6)
Aug. 91–Mar. 92	original	18.01 (0.28)	122.7 (0.9)	18.22 (0.29)	−85.7 (0.9)

Mar. 92–June 92	second	16.61 (0.82)	118.0 (2.9)	17.82 (0.63)	−91.1(2.1)

**Table 2:** M_2_ data from boreholes of various depths. The phases arc measured with respect to the local tidal potential (negative values are phase leads)

		0° azimuth		90° azimuth	
Depth (m)	Data interval	Amplitude (nrad)	Phase (deg)	Amplitude (nrad)	Phase (deg)
24	Jun. 88–Aug. 91	18.44 (0.34)	124.5 (0.6)	18.48 (0.23)	−82.8 (0.6)
36	Apr. 89–Aug. 91	21.42 (0.47)	96.5 (2.2)	14.05 (0.64)	−114.1 (2.1)
120	Dec. 89–Jul. 90	20.58 (0.81)	124.9 (2.3)	14.89 (0.82)	−91.3 (3.2)

**Table 3:** M_2_ data from multiple installations in a 36 m borehole. The phases arc measured with respect to the local tidal potential (negative values arc phase leads)

		0° azimuth		90° azimuth	
Data interval	Capsule type	Amplitude (nrad)	Phase (deg)	Amplitude (nrad)	Phase (deg)
Apr. 89–Aug. 91	original	21.42 (0.47)	96.5 (2.2)	14.05 (0.64)	−114.1 (2.1)
Aug. 91–Mar. 92	original	21.30 (0.39)	96.6 (1.1)	13.36 (0.51)	−105.6 (2.3)

Mar. 92–June 92	third	21.72 (0.37)	123.5 (1.0)	14.93 (0.41)	−93.9 (2.1)
